# Dual Mating Strategies Observed in Male Clients of Female Sex Workers

**DOI:** 10.1007/s12110-023-09439-1

**Published:** 2023-02-17

**Authors:** Jade Butterworth, Samuel Pearson, William von Hippel

**Affiliations:** 1 School of Psychology, University of Queensland, 4072 St Lucia, QLD Australia; 2 School of Business, University of Queensland, St Lucia, Australia

**Keywords:** Dual mating strategy, Male sexual preferences, Pair-bonding, Variety seeking, Sex work

## Abstract

Humans have a complex and dynamic mating system, and there is evidence that our modern sexual preferences stem from evolutionary pressures. In the current paper we explore male use of a dual mating strategy: simultaneously pursuing both a long-term relationship (pair-bonding) as well as short-term, extra-pair copulations (variety-seeking). The primary constraint on such sexual pursuits is partner preferences, which can limit male behavior and hence cloud inferences about male preferences. The aim of this study was to investigate heterosexual male mating preferences when largely unconstrained by female partner preferences. In service of this goal, female full-service sex workers (*N* = 6) were surveyed on the traits and behaviors of their male clients (*N* = 516) and iterative cluster analysis was used to identify male mating typologies. Two clusters emerged: clients seeking a pair-bonding experience and clients seeking a variety experience. Results also suggested that romantically committed men were more likely to seek a variety experience than a relationship experience. We conclude that men desire both pair-bonding and sexual variety, and that their preference for one might be predicted by fulfilment of the other. These findings have implications for relationships, providing insight into motivations for male infidelity.

Humans have complex and dynamic mating systems. Our sexual goals—or strategies—depend on who we are, where we live, and what we want at particular times in our lives. Some people desire long-term, monogamous relationships, while others prefer to have multiple short-term partners. Some people try to fulfil both of those goals simultaneously, relying on a *dual mating strategy* (Gorelik & Shackelford, 2016; Pillsworth & Haselton, 2006). A dual mating strategy (or strategic pluralism; Gangestad & Simpson 2000) involves simultaneously pursuing short- and long-term mating opportunities in different contexts, often surreptitiously (Gorelik & Shackelford, 2016; Pillsworth & Haselton, 2006).

Under the right circumstances, a dual mating strategy can combine the reproductive benefits of marriage and multiple short-term partners (Buss & Schmitt, 2019). If successful, a man can maximize his reproductive success by marrying and investing heavily in the parental care of his primary children while covertly exploiting opportunities for extramarital sex (Buss & Schmitt, 1993; Dawkins, 1986). If unsuccessful, however, he risks losing access to both his primary and affair partners (Buss, 2013; Fletcher et al., 2015). Despite these risks, infidelity rates suggest that approximately 50% of married men do attempt a dual mating strategy (Buss et al., 2017; Kinsey et al., 1953).

The greatest hurdle men face in short-term mating is finding a willing partner (Bogaert & Fisher, 1995; Hill et al., 2013). When we gather behavioral evidence regarding what men want in mating (long-term, short-term, or a combination), we can only see the mating strategies that men have employed successfully. The current study aims to circumvent this problem by investigating what men *want* when they can *have* almost anything. Observing the mating strategies employed by heterosexual male clients of female full-service sex workers allows us to bypass female partner preferences (and the limitations they impose on men’s mating strategies).

## Sexual Strategies Theory

Sexual strategies theory (Buss & Schmitt, 1993) proposes that human mating strategies are context-dependent, varying according to sex, culture, short or long-term approach, and individual and environmental factors. The theory suggests that when men and women share similar adaptive problems, their strategies will be similar, and when their adaptive problems differ, so too will their strategies (Buss & Schmitt, 2019). Multiple factors, including genetics, personality, and hormone levels, affect men’s mating strategies. For instance, men are more likely to adopt a short-term mating strategy if they are young (Buss, 2012); high in attractiveness, social status, or wealth (Buss, 1989; Clark, 2006; Gangestad & Simpson, 2000; Landolt et al., 1995); high in extraversion but low in agreeableness and conscientiousness (Schmitt & Shackelford, 2008); have a dark triad personality (high in narcissism, Machiavellianism, and psychopathy; Schmitt et al., 2017); have masculine faces (Rhodes et al., 2005)  or V-shaped torsos (Hughes & Gallup, 2003); or have high testosterone levels (Marzoli et al., 2018). With regard to the latter, testosterone levels reduce when men marry and father children (Gettler et al., 2011; Gray & Campbell, 2009), but reduce *less* in men who adopt a dual mating strategy by continuing to seek extra-pair mating opportunities (Jones et al., 2013; Welling et al., 2008).

Culture and environment also play a role in men’s mating strategies. In environments where there are more fertile women than men—for example, the hunter-gatherer Ache of Paraguay—men are more likely to engage in short-term mating (Hill & Hurtado, 1996; Pedersen, 1991). But when there are fewer women available, men are more likely to engage in long-term mating (Puurtinen & Fromhage, 2017; Schmitt, 2005). Just as pair-bonding is reflected in a desire for companionate or romantic love, men’s short-term mating is reflected in a desire for sexual variety (Buss & Schmitt, 1993; Fletcher et al., 2015; Jankowiak & Fischer, 1992). Although the integration of these pancultural desires varies according to particular cultural rules, fulfilment of one often results in forfeit of the other (Jankowiak & Paladino, 2008).

Both short and long-term mating approaches offer men distinct adaptive benefits, as well as associated costs. Pair-bonding enhances offspring survival by increasing parental investment and coalitional expansion to partner’s kin (Henrich et al., 2012). Conceptually, pair-bonding also monopolizes a woman’s reproductive resources (Buss & Schmitt, 2019), but, due to internal fertilization and paternal uncertainty, pair-bonding risks donating valuable resources to another man’s children (Marzoli et al., 2018). Similarly, there are costs associated with short-term variety seeking with multiple partners, as men attempt to maximize their mating investment while minimizing parental investment (Buss & Schmitt, 2019). To maximize number of offspring, a short-term mating strategy would be the optimal reproductive approach for men (Symons & Ellis, 1989), *if* success in securing mates is likely. Female preferences for dominant, physically attractive men as short-term mates (Bogaert & Fisher, 1995; Hill et al., 2013)—illustrated in dating apps where the top 10% of male users receive 58% of all “likes” and the lower 50% receive just 4.3% (Goldgeier, 2017)—suggest that most men are likely to fail to secure multiple short-term mates.

### Studying Men’s Mating Strategies

Because men’s mating strategies are constrained by female preferences (Bogaert & Fisher, 1995; Hill et al., 2013), their observable sexual behavior may not align with their preferred sexual behavior. To understand what men want when they are less constrained by what women want, previous research has considered men’s behavior when women’s preferences are fulfilled, lowered, or irrelevant. For instance, men who *are* what women want—that is, very high in attractiveness (as measured by fluctuating asymmetry, shoulder-to-hip ratio, and observer ratings), social status, or wealth (Buss, 1989; Gangestad & Simpson, 2000)—typically have greater sexual variety than men who are less desirable (Clark, 2006; Hughes & Gallup, 2003; Landolt et al., 1995; Rhodes et al., 2005). However, the preferences of such highly attractive men are not necessarily representative of all men because what causes men to differ in attractiveness might also make them differ in their preferences (e.g., perhaps men high in variety seeking are more likely to strive for wealth or fame in an effort to satisfy their preferences for variety).

A second approach has been to observe what men want when they are outnumbered by women in the dating pool, a situation that also results in a male preference for short-term sexual variety (Baumeister & Vohs, 2004; Hill & Hurtado, 1996; Pedersen, 1991). However, skewed sex ratios tend to emerge in unique locations and after conflict has killed many males (Guttentag & Secord, 1983). Just as these conditions shift female strategies, they may shift men’s preferences, so we cannot conclude that the preferences of men who survive or are raised in these conditions reflect those of the average man.

The mating behavior of gay men has provided further insight into men’s preferred sexual strategies when unconstrained by women. Gay men typically show a preference for short-term sexual variety, with elevated sexual infidelity (Baum & Fishman, 1994; Bonello, 2009). However, gay men may also differ from heterosexual men in their preferred sexual strategies; for example, Gonzales and Meyers (1993) found that heterosexual more than gay people pursue long-term relationships. Though each of these approaches are limited in their generalizability, they each indicate that when unconstrained by female partner preferences, men tend to be less likely to seek relationships and more likely to seek sexual variety. Thus, one conclusion that could be made from the literature is that men have a strong preference for variety and primarily form relationships in an effort to attract the best possible partner.

This conclusion, however, relies on research that often uses rates of infidelity as an indicator of variety seeking among men whose pair-bonding desires may already be fulfilled in their primary relationship. An alternative approach has been to investigate men’s sexual strategies when procuring the services of female sex workers; in the context of a paid environment, it is possible to observe whether men seek variety versus relationships independent of female preferences. Additionally, due to its transactional and discretionary nature, sex work can provide opportunities for dual mating with lower risk of discovery and expectations of future investment than other types of extramarital affairs. In studies that surveyed or interviewed male clients of female sex workers, approximately half of the participants reported a desire for companionship alongside sexual services, and approximately half reported a desire for variety of sexual partners or acts (Holzman & Pines, 1982; McKeganey & Barnard, 1996; Monto, 2010). These findings are echoed in field studies of both street- and brothel-based sex workers, who offer paid performances of not just sex, but love (Bernstein, 2010). The goal of the current research is to assess whether there are specific clusters of men who seek variety versus pair-bonding from sex workers and, if so, whether men’s relationship status is associated with cluster membership and variety-seeking behavior.

## The Current Study

To address these questions, the current study surveyed independent, high-end, internet-advertised, female, full-service sex workers about the traits and behaviors of their male clients. Although employing a sex worker is arguably an enactment of variety seeking, within this context, there are still behaviors that suggest pair-bonding and others that suggest variety-seeking. In the context of full-service sex work, a preference for pair-bonding would manifest in booking less varied sexual services (i.e., “the girlfriend experience”), a tendency to rebook the same sex worker, offers of tips and gifts, banter during booking requests, and signs of care for and kindness toward the sex worker. Conversely, evidence for variety-seeking would manifest in booking more varied sexual services (i.e., “the porn-star experience,” as well as bookings involving multiple partners).

Given the benefits associated with both pair-bonding and variety seeking, we hypothesized that even when women’s preferences are largely removed from the equation, men will seek both pair-bonding and sexual variety (Hypothesis 1). We predicted signs of pair-bonding to be more prevalent among clients who are *not* in a committed relationship (Hypothesis 2). Equally, we predicted evidence for variety seeking to be more prevalent among clients who are already in a committed relationship (Hypothesis 3). Finally, in line with previous research (Buss, 1989; Clark, 2006; Gangestad & Simpson, 2000; Landolt et al., 1995), we expected variety-seeking to be more prevalent among clients rated high in attractiveness and social skills (Hypothesis 4).

### Method

The study protocol (including sampling, materials, hypotheses, and analysis plan) was pre-registered at the Open Science Framework (OSF): https://osf.io/9m2vf/?view_only=95499f17ca1240a699ffb7ae5e891d8c. This manuscript was approved by the University of Queensland low or negligible risk review board.

### Participants

Seven independent, high-end, touring (Australia-wide), female, full-service sex workers who advertise on the same website and were acquainted with the researchers were invited via text message to participate in the study. To ensure anonymity, sex-worker demographics are not reported further than stating that all participants were Caucasian women between the ages of 20 and 35. It is worth noting in this regard that their clients do not necessarily have accurate knowledge of this information; many sex workers under-report their age and weight, and over-report their education to their clients. Inclusion criteria required participants to: (a) have engaged in paid sex work within the last 12 months (to maximize accurate recollection); (b) have charged a minimum of $600/hour for a standard booking (to minimize risk of eliciting traumatic memories given the increased rate of violence experienced by lower-income sex workers; Shaver 2005); and (c) be able to provide data for approximately 100 clients. One of the seven participants was excluded from the study for failing to meet these criteria and as such, her data were removed prior to analysis (but are available in the data file on the OSF). From the remaining six participants, data for 516 clients were collected for analysis. This sample size did not reach our pre-registered minimum goal of 600 clients, but further recruitment efforts proved unsuccessful.

### Measures

The 15-question survey first requested client demographics of age, ethnicity, and relationship status. To measure relationship status, participants were asked, “Is the client in a relationship?” To measure repeat visits, participants were asked, “Has the client booked you more than once?” For booking type we asked, “What service did the client book?” (industry standard includes girlfriend experience, porn-star experience, or couples—which includes any scenario involving one man and more than one woman). For banter, “Has the client attempted casual conversation with you via text?” For tips and gifts, “Has the client given you gifts or tips?” To measure signs of care for the sex worker, participants were asked, “Do you think the client cares about you and your enjoyment?” Sex worker ratings of client traits were measured on a 10-point response scale from 1 (not at all) to 10 (very much), for the questions: “How attractive was the client?” “How kind was the client?” and “How socially skilled was the client?” Additionally, to measure location, duration, and fee of the booking, participants were asked, “Did the client visit your in-call location or did you visit their hotel/home?” “What was the duration of your most recent booking with the client?” and “What was the fee for the client’s most recent booking?” As an exploratory variable, participants were also asked, “Do you think the client would be embarrassed to request their preferred sexual acts in a non-paid environment (such as on a date or with their partner)?” which we coded as our indicator of kink.

### Procedure

Participants were met at a public location of their choosing and given the Participant Information Sheet and Consent Form to read and sign. Participants were then asked to access their text messages with clients and, starting with the most recent client, provide oral responses to the 15 survey questions, which were recorded in a spreadsheet. This process was repeated for as many unique clients as possible within a two-hour period.

## Results

### Preliminary Analyses

Client demographics, location of the booking, fees, etc., are provided in Table 1. Consistent with Hypothesis 1, scores were evenly distributed across all focal variables, indicating that men display evidence for both pair-bonding and variety-seeking when largely unconstrained by female mating preferences (Table 1). Note that the data suggest that 44% of the men are in a relationship, but there are two caveats to this finding. First, we are missing data on 20% of the men, as the sex workers were unaware of whether these clients were in a relationship. Second, although sex workers often noted that they had clear evidence that their clients were in a relationship (e.g., when the booking was in a man’s home and his wedding photos were on the wall; when men talked about their wife, etc.), sometimes they indicated that their clients were single but acknowledged that this belief was based on the clients’ claims and that the men might have been lying.

**Table 1:** Descriptive statistics

Variable	Valid %	*M* (*SD*)
In a Relationship	44	
Repeat	59	
Girlfriend Experience	56	
Banter	64	
Care	39	
Gifts	29	
Kink	20	
Incall Location	56	
Caucasian	76	
Asian	12	
Other Ethnicity	12	
Attractive		4.94 (2.20)
Kind		6.02 (2.13)
Social Skill		5.73 (2.30)
Age		39.26 (11.77)
Duration (hrs)		2.76 (5.11)
Price (AUD)		1722.29 (1705.84)

*Note*. Due to missing data, valid percentage for relationship status includes 419 of 516 total clients

As an initial test for evidence for pair-bonding within a paid environment, Pearson’s *r* and point biserial correlations were conducted across the various measures (Table 2). Due to the number of tests included in these analyses, we only highlight those results here that achieved a critical *p* value of 0.001. These analyses showed that a tendency to rebook the same sex worker was positively associated with banter, gifts, and kindness (Table 2). A preference for the “girlfriend experience” was positively associated with care and negatively associated with kink. Lastly, clients in a committed romantic relationship were more likely to book the “porn-star experience” and less likely to engage in banter or display care toward the sex worker. All of these preliminary results are consistent with hypotheses.

**Table 2:** Correlations among focal, clustering variables

Variable	Relationship	Repeat	Experience	Banter	Care	Gifts	Attractive	Kind	Social Skill
Relationship	—								
Repeat	0.08	—							
Experience Type	0.26***	0.06	—						
Banter	−0.17***	0.30***	−0.02	—					
Care	−0.22***	0.13**	−0.15***	0.39***	—				
Gifts	0.04	0.30***	0.11*	0.32***	0.32***	—			
Attractive	0.04	−0.10*	−0.08	0.05	0.11*	−0.02	—		
Kind	−0.10*	0.15***	−0.12**	0.27***	0.46***	0.26***	0.25***	—	
Social Skill	0.16**	0.17***	−0.01	0.15***	0.17***	0.19***	0.40***	0.45***	—
Kink	0.07	0.10*	0.24***	0.07	0.02	0.15***	−0.11*	−0.08	−0.06

*Note*. Relationship, Repeat, Banter, Care, Gifts, and Kink were dichotomously coded (no = 0, yes = 1). Experience was dichotomously coded (girlfriend experience = 1, porn-star experience = 2)

* *p* < .05, ** *p* < .01, *** *p* < .001

### Primary Analysis

Iterative partitioning cluster analysis was conducted in the statistical program R (version 4.1.1) using the cluster package (Shendre, 2020) to identify client typologies. To account for mixed data, (dis)similarity of data points was measured using Gower distance—a metric able to calculate distances between individuals when attributes have been measured on both categorical and continuous scales (without assigning more weight to binary-coded categorical variables; Martin 2016). The partitioning around medoids clustering algorithm was implemented, as it is most appropriate for Gower distance, robust to noise and outliers, and allows for easier interpretation (as cluster centers are observations rather than means/centroids; Martin 2016; Shendre, 2020).

The number of clusters extracted (two) was determined a priori, to test the hypothesis that clients would cluster based on their preference for variety or pair-bonding. The silhouette coefficient (which compares the average distance among observations within the same cluster to the average distance among observations in other clusters) was used to extract the optimal number of clusters to validate the a priori decision (Filaire, 2018). The following variables were entered into Model 1: relationship status, repeat, experience, banter, care, gifts, attractiveness, kindness, social skills, and kink. To determine if the clusters were robust, the analysis was then rerun after removing the less important variables (Aldenerfer & Clashfield, 1984; Jolliffe et al., 1995). Specifically, the variables attractiveness, kindness, social skills, and kink were removed from Model 2, which was also validated using the silhouette coefficient.

**Fig. 1:**
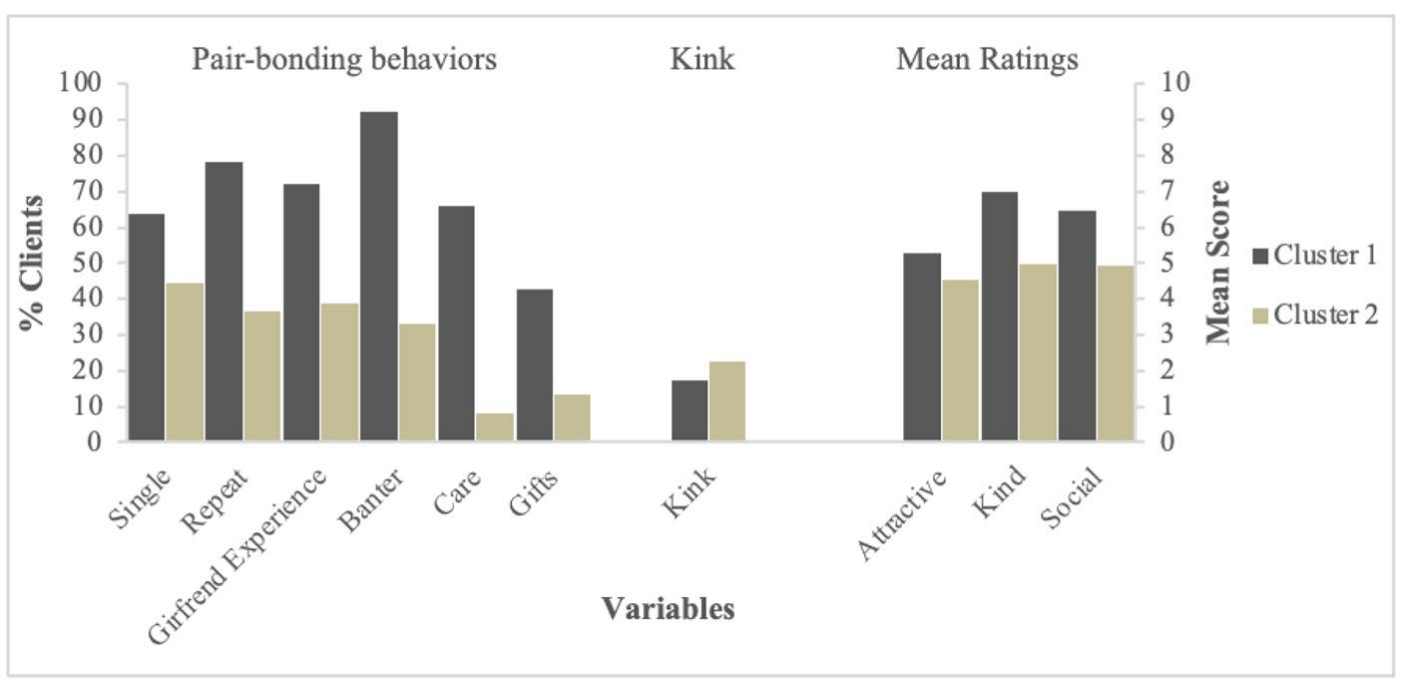
Model 1 cluster summary displaying percentage of clients engaging in pair-bonding behaviors, requesting kink services, and their mean scores on continuous variables

Figure 1 displays within each cluster for Model 1: the percentage of clients who show evidence of pair-bonding behaviors (as opposed to variety-seeking) and their mean scores on attractiveness, kindness, and social skills as rated by their sex worker. Clients in cluster one—which we label as men seeking a *pair-bonding experience*—were primarily single men who tend to rebook the same sex worker, prefer the “girlfriend experience,” engage in banter, show care for the sex worker, provide tips and gifts, and were rated as above average in attractiveness, kindness, and social skills. This cluster provides preliminary support for Hypothesis 2, in that evidence for pair-bonding was more prevalent among clients who are *not* in a committed relationship.

Conversely, clients in cluster two—men seeking a *variety experience*—were characterized as primarily men in a committed romantic relationship who prefer the “porn-star experience” and were rated as below average in attractiveness, kindness, and social skills. This cluster provides preliminary support for Hypothesis 3, in that evidence of variety-seeking was more prevalent among clients who *are* in a committed relationship. The structure of the clusters—dominated by either pair-bonding or variety-seeking behaviors—lends further support to Hypothesis 1, in that men appear to seek both pair-bonding and variety when unconstrained by women’s preferences.

Inconsistent with Hypothesis 4, evidence of variety seeking was not more prevalent among clients rated high in attractiveness or social skills. Neither cluster was more likely to display a proclivity for potentially embarrassing or unusual sexual activities. Rerunning the cluster analysis after removing the “kink” variable resulted in no change in the clusters.

### Cluster Validation

**Fig. 2:**
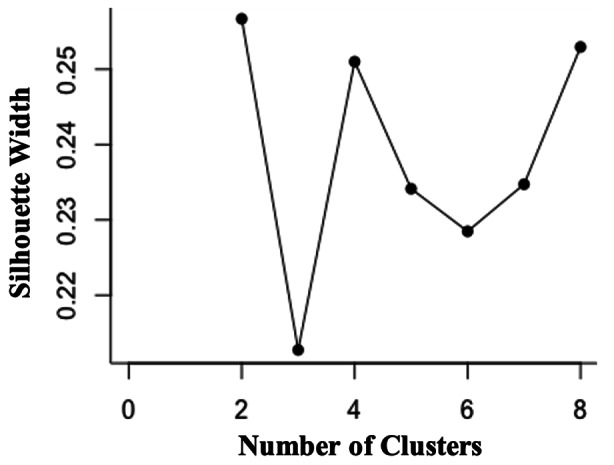
Silhouette width as a function of number of clusters for Model 1

**Fig. 3:**
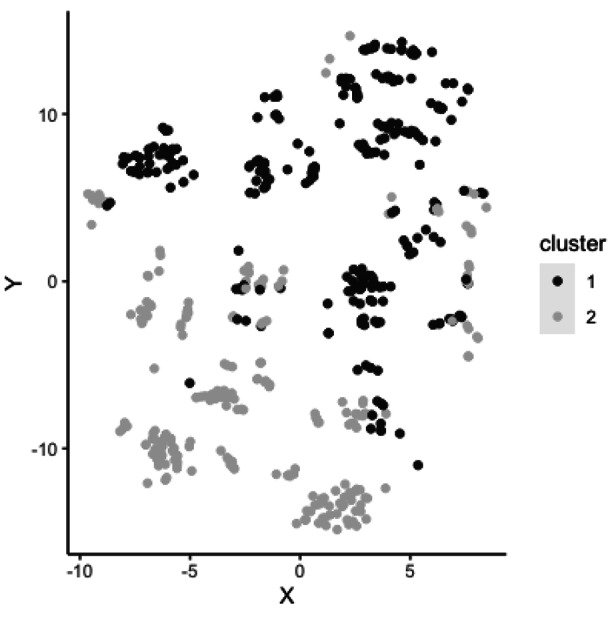
Visualization of cluster groups in a two-dimensional plot using t-distributed stochastic neighborhood embedding (t-SNE)

Silhouette width was calculated for Model 1 segmentation options ranging from two to eight clusters and, as can be seen in Fig. 2, the width was maximized (indicating the optimal number of clusters) at two, supporting the a priori decision to extract two cluster groups from the data. Though the four and eight cluster solutions displayed similar width, they were less preferred than the more parsimonious two-cluster solution (Filaire, 2018). The optimized two-cluster solution, containing the cluster structures noted above, further supports Hypothesis 1; when largely unconstrained by women’s preferences, men still seek both pair-bonding and sexual variety. Visualization of these two clusters is provided in Fig. 3.

**Fig. 4:**
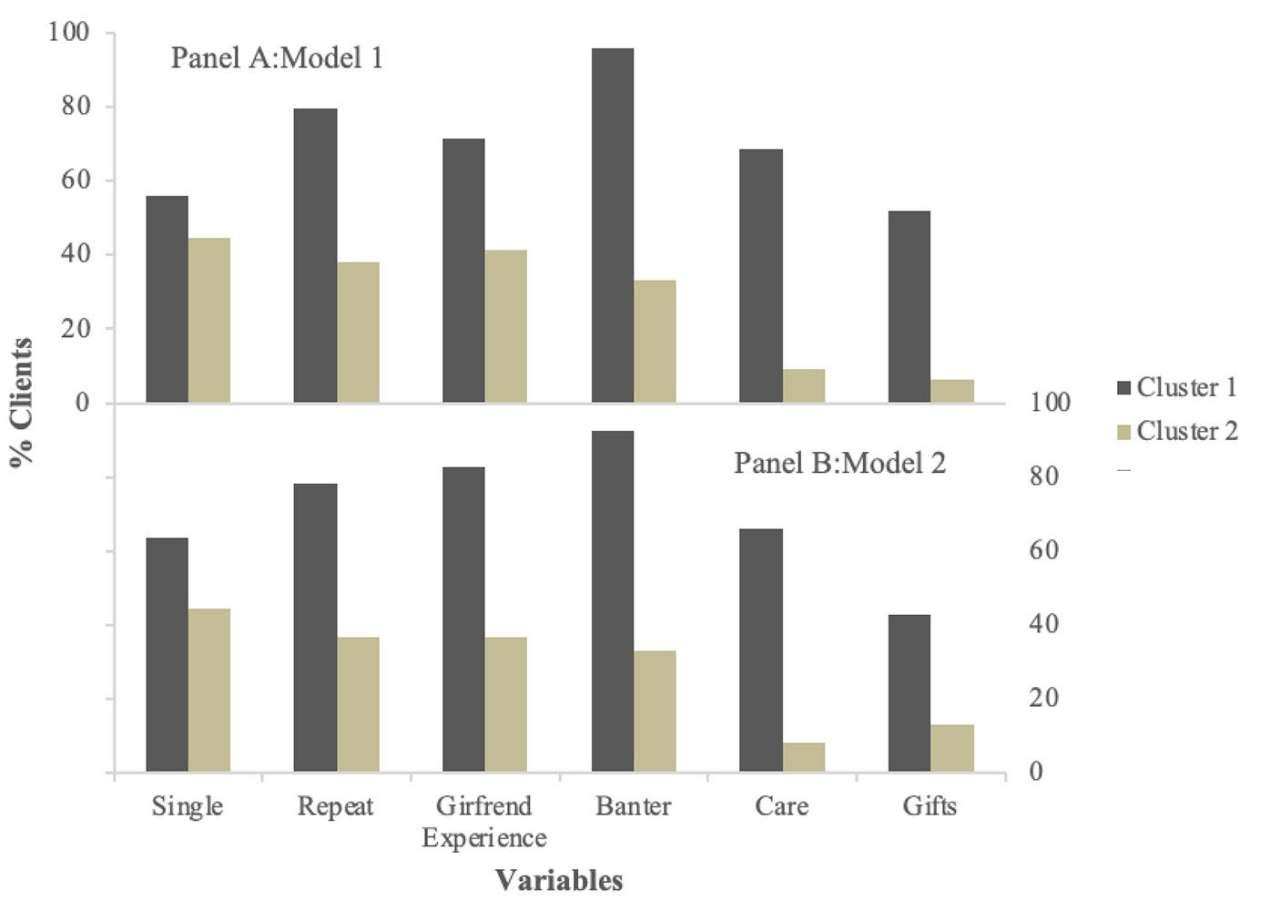
Percentage of clients who are single and who are engaging in pair-bonding behaviors as a function of cluster membership for Model 1 (Panel A) and Model 2 (Panel B)

Robustness of clusters was tested in Model 2 by rerunning the analysis with fewer variables. As can be seen in Fig. 4, the extracted clusters in both models were characterized by the same patterns of behavior seen in Model 1. That is, clients seeking a *pair-bonding experience* were characterized as primarily single men who rebook the same sex worker, prefer the “girlfriend experience,” engage in banter, show care for the sex worker, and provide tips and gifts, with the opposite pattern visible in men seeking a *variety experience*. Silhouette width calculated for two to four clusters (with reduced scope due to the reduced number of variables) maximized at four (width = 0.38), however two clusters provided a simpler model with only a slight loss in width (width = 0.36) that was also superior to a three-cluster solution (width = 0.34), and thus the two-cluster solution was again retained (Filaire, 2018).

### Controls for Sex Worker

To assess if the extracted typologies were driven by differences among sex workers rather than differences among clients, we performed a chi-square test to examine if cluster membership varied as a function of sex worker. The relationship between these variables was significant, χ^2^(5, *N* = 516) = 48.23, *p* < .001, indicating that cluster group membership varied as a function of sex worker. See Table 3 for cross-tabulation counts.

**Table 3:** Cross-tabulation counts of cluster membership as a function of sex worker

Sex Worker	Cluster 1	Cluster 2	Total
1	42	28	70
2	68	32	100
3	38	40	78
4	52	36	88
5	49	31	80
6	24	76	100
Total	273	243	516

Next, we conducted a series of regression analyses to investigate whether cluster membership predicted client scores on pair-bonding/variety-seeking behaviors when controlling for sex worker. We used binary logistic regression for categorical dependent variables (relationship status, repeat, experience, banter, care, and gifts), and ordinary least squares regression for continuous dependent variables (attractiveness, kindness, and social skills). To control for sex worker, we dummy coded the first five sex workers as yes/no (as such, the sixth sex worker was determined by dummy codes on the first five). We included cluster membership and the five dummy-coded sex workers as predictors in all models. As can be seen in Table 4 and consistent with Hypothesis 1, when controlling for sex worker, cluster membership remains predictive of client scores, suggesting that the extracted typologies are driven by differences among clients, in addition to any variance accounted for by individual sex workers.

**Table 4:** Predictive ability of cluster while controlling for sex worker in binary logistic and linear regression analyses

Dependent Variable	*B* (*SE*)	Wald χ^2^	*p*
Relationship	−0.66 (0.22)	8.90	0.003
Repeat	2.64 (0.29)	85.19	< 0.001
Experience	−1.57 (0.22)	49.32	< 0.001
Banter	3.26 (0.29)	126.69	< 0.001
Care	4.17 (0.45)	85.54	< 0.001
Gifts	1.75 (0.26)	45.77	< 0.001
	β	*t*	*p*
Attractive	−0.20	−4.47	< 0.001
Kind	−0.49	−12.15	< 0.001
Social Skills	−0.37	−8.80	< 0.001

*Note.* All nine models include cluster and the five dummy coded sex worker variables as simultaneous predictors. The results displayed above are for the predictor “cluster” only

Finally, to assess whether cluster membership predicted client scores on the various pair-bonding/variety-seeking behaviors when clients were nested within sex worker, we used the lme4 package (Pinheiro et al., 2022) in the R statistics program to fit linear mixed-effects (multilevel) models to the data. As seen in Table 5, when clients were nested within sex worker, cluster membership predicted whether clients would rebook the sex worker, book the girlfriend experience, engage in banter, show signs of care for the sex worker, provide tips or gifts, and be rated high in kindness and social skills by the sex worker. However, cluster membership no longer predicted client relationship status (or, less importantly, attractiveness ratings) when clients were nested within sex worker. An analysis of the individual items within clusters revealed that relationship status did not predict repeat bookings, banter, tips or gifts, but relationship status continued to predict whether clients chose the girlfriend experience (*z* = 2.889, *p* = .004) and whether they showed signs that they cared for the sex worker (*z* = − 2.212, *p* = .027) in these nested models.

**Table 5:** Predictive ability of cluster membership while controlling for sex worker in multilevel models

Dependent Variable	β [95% CI]	*p*
Relationship	0.15	0.138
	[− 0.02, 0.33]	
Repeat	−0.43	< 0.001
	[− 0.52, − 0.34]	
Experience	0.31	0.001
	[0.21, 0.40]	
Banter	−0.55	< 0.001
	[− 0.68, − 0.43]	
Care	−0.50	0.001
	[− 0.65, − 0.36]	
Gifts	−0.27	0.003
	[− 0.39, − 0.16]	
Attractive	−0.81	0.14
	[− 1.72, 0.10]	
Kind	−2.05	0.001
	[− 2.58, − 1.51]	
Social	−1.68	< 0.001
	[− 2.22, − 1.15]	

*Note.* Since residuals of most models were skewed, the relationships between cluster membership and client scores were also modelled with binomial or ordinal mixed effects regression (see OSF: https://osf.io/9m2vf/?view_only=95499f17ca1240a699ffb7ae5e891d8c for details). No change to parameter significance was observed

## Discussion

The aim of this study was to investigate men’s mating preferences when largely unconstrained by women’s preferences, by examining the traits and behaviors of male clients of female full-service sex workers. We predicted that in a paid (and therefore, largely unconstrained) mating environment, men would seek both pair-bonding and sexual variety (Hypothesis 1), and that their individual preferences could be predicted by their relationship status (Hypotheses 2 and 3).

In line with Hypothesis 1, we found two distinct typologies of clients—those seeking a pair-bonding experience, and those seeking a sexual variety experience. In line with Hypothesis 2, preliminary evidence suggested that pair-bonding behaviors with the sex workers was more prevalent among single men, but this result did not hold up at the cluster level when clients were nested within sex worker. Conversely, and in line with Hypothesis 3, evidence for variety-seeking (a preference for the “porn-star experience”—sometimes involving multiple partners) was more prevalent among men who were already in a committed relationship, and this finding emerged in the nested analyses as well. Finally, Hypothesis 4 predicted that men seeking variety would be rated high in attractiveness and social skills by the sex worker, and this prediction was not supported.

### Limitations

An important consideration is the degree to which clients of full-service sex workers are representative of the male heterosexual population. Sex work is often referred to as the “oldest profession” because its occurrence has been recorded globally across every period in history, but it is only consumed by a fraction of men (García et al., 2017; Pitts et al., 2004). For example, an Australian survey found that 23% of men had paid for sex at least once in their lives (Pitts et al., 2004). These commercial sex clients reported that their consumption was primarily motivated by ease of access, which is consistent with findings that prostitution rates increase in societies that hold oppressive attitudes towards sex. Where casual sex is readily available, the demand for commercial sex declines (García et al., 2017). Along this line, it is also important to note that we only examined clients of *independent, high-end* sex workers (minimum rate of $600/hour), which restricted our client sample to men of high socio-economic status, thus reducing the generalizability of results.

The small number of sex workers from whom data was collected is another important consideration, in part because it did not allow us to reach our pre-registered minimum number of clients. Additionally, each of the six sex workers provided data for up to 100 clients, which sometimes meant reporting bookings that occurred over 12 months in the past and hence were not well-remembered. A greater number of sex workers would have allowed for collection of more recent data for a greater number of clients and thereby increased confidence in the accuracy of their reports, particularly the issue of whether their clients were in a relationship. As noted above, there was a great deal of missing data on this variable, and sex workers also noted that they were sometimes unsure if their clients were telling the truth when they claimed to be single. We also had no way of assessing the quality of men’s relationships in our data collection, and hence it was impossible to ascertain if the pair-bonding needs of men who were in relationships were satisfied by those relationships.

Finally, we have interpreted these clusters as evidence that some men seek variety and some men seek relationships when they pay for sex. It is important to keep in mind, however, that these findings are susceptible to all the alternative explanations that emerge in non-experimental data. For example, it is possible that we failed to measure some key variables about the clients or sex workers that would have accounted for variance in cluster membership or any of the individual items. Thus, future research is necessary to replicate and extend the current findings.

Future research could expand this study in two ways. First, research could include a broader range of sex workers (independent, agency-contracted, and brothel-employed) while relaxing the exclusion criteria to include a broader range of sex work income (and therefore a broader range of clientele). This expansion would increase the robustness and generalizability of the findings. Second, research could extend the scope of this study by collecting demographics as well as physical and personality characteristics of the *sex workers*. This information would allow future research to determine which female attributes attract men who are seeking pair-bonding versus variety and might align those findings with existing literature. For instance, men value intelligence in a long-term mate (Goetz et al., 2012; Kenrick et al., 1993), but look for cues related to sexually permissive attitudes in a short-term mate (Campbell et al., 2009; Fink & Penton-Voak, 2002). Consistent with these differences, many sex workers adapt their business strategies according to client, venue, and culture in an effort to maximize their income (Brennan et al., 2004; Yu et al., 2018). Follow-up research might consider the impact of such strategies among sex workers on dual mating strategies of their clients. The differences in cluster membership found across the six sex-workers in Table 3 is consistent with the possibility that they had different business strategies or features that led their clients to choose one type of experience over another.

## Conclusion

Through investigation of men’s mating preferences and strategies when largely unconstrained by women’s preferences, we found that men sought both pair-bonding and sexual variety. We also found that their preference for one was at least partially influenced by fulfilment of the other—husbands were more likely than single men to choose the sexual experience that reflects variety (the porn star experience) over relationships (the girlfriend experience). These findings are consistent with the presence of dual-mating strategies in men and highlight the fact that men seek both sexual variety and pair-bonding. Importantly, these findings also clarify that men seek relationships in service of their own needs, not only to satisfy their female partners.

## Data Availability

Data and materials are available through the Open Science Framework via the following link: https://osf.io/9m2vf/?view_only=95499f17ca1240a699ffb7ae5e891d8c.

## Code Availability

R-Markdowns of coding for cluster analysis and multilevel modelling are available through the Open Science Framework via the following link: https://osf.io/9m2vf/?view_only=95499f17ca1240a699ffb7ae5e891d8c.
